# Association of single Nucleotide Missence Polymorphism Val109Asp of Omentin-1 gene and coronary artery disease in Pakistani population: Multicenter study

**DOI:** 10.12669/pjms.335.13110

**Published:** 2017

**Authors:** Shazia Nazar, Sitwat Zehra, Abid Azhar

**Affiliations:** 1 Dr. Shazia Nazar, MBBS. Demonstrator, Department of Physiology, Karachi Medical and Dental College, Karachi, Pakistan; 2 Dr. Sitwat Zehra, Ph.D. Assistant Professor, Dr. A. Q. Khan Institute of Biotechnology and Genetic Engineering (KIBGE), University Of Karachi, Karachi, Pakistan; 3 Dr. Abid Azhar, Ph.D. Director General, Dr. A. Q. Khan Institute of Biotechnology and Genetic Engineering (KIBGE), University Of Karachi, Karachi, Pakistan

**Keywords:** Coronary artery disease, Genotype, Omentin-1, Polymorphism

## Abstract

**Background & Objective:**

Coronary artery disease (CAD) is a most important cause of morbidity and mortality worldwide as well as in Pakistan. Recent studies have shown that the combination of obesity, insulin resistance and fluctuation in circulating adipocytokines levels is associated with the pathogenesis of coronary artery disease. Omentin-1 is recently found adipocytokine that is highly expressed in visceral adipose tissue. It has anti- inflammatory properties and is negatively correlated with ischemic heart disease. Therefore, this study was designed to investigate the relationship between omentin-1 Val109Asp polymorphism and CAD in Pakistani population.

**Methods:**

A total of 350 subjects were included in the study. Two hundred fifty were diagnosed with coronary artery disease while 100 served as healthy controls. PCR-RFLP was performed at Dr. A Q. Khan Institute of Biotechnology (KIBGE) to analyze Val109Asp polymorphism. In this, case control study SPSS software version 16 (Chicago, IL, USA) was used for data analysis. Continuous variables and categorical variables were presented as mean±SD or in percentage. Independent sample test and chi-square test was performed to compare the differences in means between cases and controls. Genotype distribution was analyzed by chi-square test and results were presented as percentage and frequency. Multivarible regression analysis indicated that Val109Asp SNP might be an independent risk factor for CAD susceptibility after adjustment for some well- known CAD risk factors including age, gender, body mass index, smoking, hypertension, diabetes mellitus and lipid abnormalities. There was estimation of odd ratios (OR) and 95% confidence intervals (CIs) to determine the correlation between genotypes and the risk of CAD. (p> 0.05). Genotype frequencies were compared by Chi-square test.

**Results:**

There was prevalence of Omentin-1 Val109Asp polymorphism in both case and control groups. However, Val/Asp (heterozygous mutant) genotype was detected more frequently in patients with CAD, OR(95%)=1.921; CI=1.173-3.1469 in comparison of Asp/Asp and Val/Val genotypes.

**Conclusion:**

Individuals having Val/Asp heterozygous gemotype of omentin-1 gene polymorphism are at more risk of developing CAD in Pakistani population, further studies are required in different populations and ethnicities to confirm our findings.

## INTRODUCTION

Coronary artery disease (CAD) is emerging as the principal killer of the 21st century in both developed and developing countries. Globally 17.4 million deaths, that is29% of all deaths occur due to CAD, per year.^1^ The prevalence of CAD in Pakistan is about 6%.^2^,^3^ Atherosclerosis is the single most significant cause of CAD. It is characterized by chronic inflammation of arteries with development of fatty plaque in arterial wall, leads to ischemia of heart, brain and extremities.^4^ Inflammation is caused by several hormones like proteins called adipokines, released from adipose tissues, which now, considered as main endocrine tissue of the body.^5^ Omentin-1 is a recently identified, visceral adipose tissue derived adipokine consists of 295 amino acids, coded by gene located at chromosome 1q 21-23.^6^,^7^ Omentin-1 is reported to be highly expressed in epicardial adipose tissue (EAT) around the heart and coronary arteries.^8^ Omentin-1 is considered as an anti- inflammatory adiopokine.^9^

Recent studies have shown that Serum levels of omentin-1 significantly reduced in coronary heart disease and associates negatively with markers of atherosclerosis including: body mass index (BMI), waist circumference, HDL and hsC-reactive protein. So, omentin-1 may serve as a novel biomarker for early detection of CAD.^10^,^11^ Single gene missence polymorphism in exon 4 of omentin-1 gene was identified by Scaffler in 2007 by random sequencing and made known that nucleotide +326 is polymorphic A/T. However; significance of missense polymorphism Val109Asp in the human Omentin-1 gene in connection with CAD has not been studied yet in Pakistan.

This study aimed to establish local perspective as there is paucity of local data and was designed to determine whether omentin-1 gene polymorphism is a predisposing factor in development of CAD in Pakistani population because coronary artery disease and its management is emerging as a great challenge to deal with, hence, data collected from this study will help in establishing the role of omentin-1 in pathophysiology and therapy of CAD.

## METHODS

This study was approved by the ethical review board committees of concerned institutions. A total of 250 patients were enrolled in this study between the period of January 2016 to April 2016 in Karachi Institute of Heart Disease (KIHD), Civil Hospital Karachi (CHK) and Darulsehat Hospital Karachi. All patients had undergone coronary angiography. CAD was defined as more than 50% obstruction of one or more coronary arteries. Severe chest pain, ST segment elevation and T wave inversions in more than two leads in ECG, elevated Troponin-I >0.01ng/ml) and positive stress test for ischemia were indications of angiography. The acute infections, malignancy, valvular heart disease, liver disease (ALT >59 units/L) and renal disorders (Creatinine >1.2 mg/dl) were excluded from the study. History of risk factors such as diet, lack of exercise, smoking, family history of cardiac disease, hypertension, diabetes mellitus, and hypercholesterolemia was recorded. Age and gender matched 100 controls were included in this study. Controls were defined as individulas had negative exercise tolerance test or less than 50% stenosis of coronary arteries on angiography, moreover, those control subjects who reported arthritis, diabetes, renal disorders and history of recent infections were excluded from the study. All participants were informed about the methods and benfits of this medical research and their informed consents were taken.

### Clinical and laboratory measurements

The history of disease was recorded in detail, Body mass index of all participants were calculated as kg/m^2^. Waist circumference was measured between upper margins of iliac crests and lower border of rib cage. Morning blood samples were collected in EDTA tubes after 12 hour overnight fasting. Routine lab tests including serum cholesterol, triglycerides (TC), high density lipoprotein cholesterol (HDL-C), low density lipoprotein cholesterol (LDL-C), fasting blood sugar (FBS), serum creatinine were analyzed by an automated analyzer (Olympus Irish branch) in hospital laboratory.

### DNA Extraction

Genomic DNA was extracted from whole blood via salting out extraction method (Miller *et al.*,1988). Extracted DNA was quantified by Nano drop (Thermo scientific USA). The integrity of DNA was checked by resolving 3ul of genomic DNA in 0.8% agarose gel in horizontal gel electrophoresis.

### PCR Analysis

For amplification of polymorphic axon 4 of omentin-1 gene having Val109Asp polymorphism, Polymerase chain reaction was performed (Mullis et al., 1987) by the set of F-primer 5′-AATCAGAAGGCAGTCCTCCC-3′ and the R-primer 5′-TCGGGGAGCACAGAGTGTAC-3′ (Schaffler et al., 2007). For PCR, total 50μl volume was prepared containing genomic DNA, 0.2 mM dNTPs, 1X PCR buffer of pH-8.3, 1.5mM MgCl_2_, 5 units of Taq DNA polymerase. PCR amplification was carried out according to the program conditions with initial genomic DNA pre-denaturation at 94°C for 5 minutes followed by 35 cycles consisting of 60minutes denaturation at 94°C, 40second annealing at 62°C, and 60 second extension at 72°C. The final extension included 5minutes at 72°C. Amplified PCR product of 471 bp was resolved on 2% of agarose gel and visualized on gel doc.

**Fig.1:**
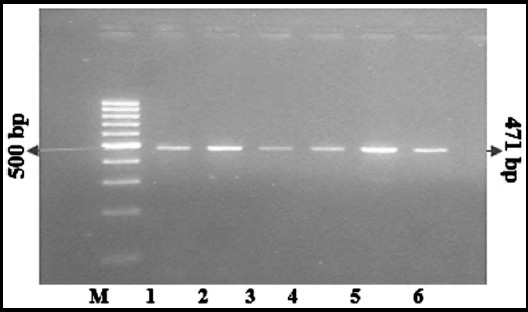
Lane 1-6 PCR product of omentin-1 gen (471 bp) M=DNA molecular weight marker (100 bp)

**Fig.2:**
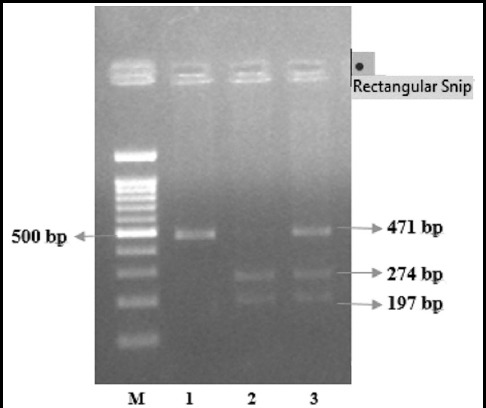
PCR-RFLP analysis of +326 A/T polymorphism of omentin-1 gene, M=DNA MW marker, 1 = AA homozygous, 2 = TT homozygous, 3 = AT heterozygous.

### Restriction Enzyme Analysis

PCR products were purified and treated with 10U of *AccI* restricyion enzyme which recognized +326A/T polymorphic site and cut it into two fragments of 274 and 197 bps(Molecul-On, Germany). The digested products were visualized on 2.5% of agarose gel. Individuals with Val/Asp (GTC/GAC) genotype had 471bp, 274 bp and 197 bp fragments, subjects with Asp/Asp(GAC/GAC) genotype had 471 bp fragments and subjects with Val/Val (GTC/GTC) showed two fragments of 274 bp and 197 bp.

### Statistical analysis

In this study SPSS software version 16 (Chicago, IL, USA) was used for data analysis. Continuous variables and categorical variables were presented as mean ± SD or in percentage. Independent sample t-test and chi-square test was performed to compare the differences in means between cases and controls. Genotype distribution was analyzed by chi-square test and results were presented as percentage and frequency. Multivariate logistic regression analysis was performed with adjustment for age, BMI, waist circumference and the prevalence of smoking, hypertension, diabete and family history of CAD. The association of AT genotype and CAD was estimated using dominant model (defined as AT vs. TT+AA) and recessive model (defined as TT+AT vs. AA), respectively. Odds ratios (OR) with their 95% confidence intervals (CI) were reported. A multivariate logistic regression analysis was used for association analyses with adjustments for age, gender, BMI, smoking, hypertension, and diabetes mellitus. P-value<0.05 was considered statistically significant. There was estimation of odd ratios (OR) and 95% confidence intervals (CIs) to determine the correlation between genotypes and the risk of CAD (p> 0.05).

## RESULTS

In current study, 250 patients diagnosed with CAD (92 females and 158 males with a mean age 51.39 ± 6.3 years) and 100 control subjects with normal blood vessels (21 females and 79males with mean age 49.78 ± 6.4) were recruited. The demographic, clinical and anthropometric parameters of the both case and control groups are shown in Table-I. There is no significant difference between two groups in term of age, gender, height, weight, total cholesterol, LDL and triglycerides levels. However, some parameters including: waist circumference (39.9±3.1 vs 31.9± 3.75 inches, p<0.05), HDL levels (25.9± 6.27 vs 42.38±10.03 mg/dl, p<0.05), FBS (154±64 vs 88.23±13.44, p<0.05) in patients were determined to be significantly higher as compared to the healthy controls. Moreover, patients of CAD had strong family history of hypertension (54% vs 9%, p<0.05) and heart diseases (57% vs 34%, p<0.05). Omentin-1 gene PCR-RFLP analysis revealed that out of 250 patients, 81 were homozygous for the Asp/Asp genotype, 22 were homozygous for the Val/Val genotype and 147 were heterozygous for the Val/Asp genotype. In control group, 62 participants had Asp/Asp homozygous genotype, 7 participants had Val/Val homozygous genotype and 30 participants had Val/Asp heterozygous genotype. The allelic frequency of both study groups were significant different. Frequencies of Asp109Asp, Val109Asp and Val109Val genotypes among patients with CAD were 32%, 58% and 8.8% respectively, that was (Asp109Asp - 62%, Val109Asp- 30% and Val109 Val - 7%) (Table-II). The study group was further analyzed according to the genotype groups. The anthropometric, clinical and metabolic variables were not significantly different in cases and controls, except there was a significant male predominance in the Val/Asp genotype. Moreover, the individuals with genotype Val/Asp had strong family history of CAD (Table-III). Since CAD patients had higher frequency of abnormality in serum levels of HDL-C and blood glucose and were found with strong history of cardiac disease in family and hypertension as compared to controls, so, multivariate logistic regression analysis was used to exclude the effect of confounding factors on genetic association. AT genotype still demonstrated the strong association with CAD in our samples after adjustment for those well-known CAD risk factors including age, gender, BMI, smoking, hypertension, diabetes mellitus, lipid abnormalities suggesting that AT genotype might be an independent risk factor for CAD susceptibility (Table-IV).

**Table-I:** Comparison of clinical, demographic and anthropometric characteristics of patient and control groups.

*Variables*	*CAD (n=250)*	*Control (n= 100)*	*P-value*
Age, years	51.3 ± 6.38	49.7± 6.4	NS
Gender, Males Females	63.2 % 36.8 %	71% 29%	NS
BMI, kg/m^2^	28.5± 8	25± 4	NS
Waist circumference, inches	39.95 ± 3.1	31.95 ± 3.7	0.003
Systolic blood pressure, mm Hg	135 ± 18	128 ± 7	NS
Diastolic blood pressure	90 ± 13	84 ± 6	NS
FBS, mg/dl	154 ± 4	88 ± 13	0.05
Cholesterol, mg/dl	239 ± 25	230± 21.8	NS
Triglycerides, mg/dl	132.3 ± 40	131 ± 32	NS
HDL, mg/dl	25.9 ± 6.2	42.3 ± 10	0.003
LDL, mg/dl	126 ± 85	123 ± 6	NS
HTN	54 %	9%	0.001
Smokers	45 %	55 %	NS
Family history of CAD	57.2 %	34 %	0.034
History of diabetes mellitus	34 %	9 %	0.05
Exercise, yes	32 %	37 %	NS
Junk food intake	55 %	57 %	NS

Continuous data is presented as mean± SD, categorical parameters are describes as percentage. P values for independent t test and chi-square tests less than 0.05 is significant. NS= non-significant.

**Table-II:** Comparison of genotypes of omentin-1 gene Val109Asp in patients with CAD and control group.

*Genotype*	*CAD (n=250)*	*Control (n= 100)*	*Odd ratios (CI= 95%)*	*P-value*
Asp/Asp	(81) 32 %	(63) 63%	0.335 (0.2241-0.5635)	0.05
Val/Asp	(147) 58 %	(30) 30 %	1.921 (1.173-3.1469)	0.009
Val/Val	(22) 8.8%	(7)7 %	1.008 (1.231-1.222)	NS

Genotype distribution is presented as proportion/percentage. CI =confidence interval. NS= non-significant. Asp/Asp= GAC/GAC; Val/Asp= GTC/GAC; Val/Val= GTC/GTC

**Table-III:** Comparison of clinical parameters of study group to the genotypes.

*Variables*	*Asp/Asp (n=143)*	*Val/Asp (n=177)*	*Val/Val (n=29)*	*P-value*
Age, years	52 ± 7.7	53 ± 4.8	51 ± 9.8	NS
Gender, Male Female	72 71	144 65	23 6	0.04
Diastolic blood pressure, mm Hg	85 ± 4.48	90 ± 2.3	84 ± 7	NS
Systolic blood pressure, mm Hg	130 ± 59	145 ± 20.4	135 ± 30.06	NS
BMI, kg/m2	28.5 ± 2.67	26.7 ± 3.8	28 ± 5.9	NS
Waist circumference, inches	35 ± 7.3	38.8 ± 3	36 ± 6.8	NS
HDL, mg/dl	42 ± 6.25	41 ± 2.89	39 ± 6.9	NS
Cholesterol, mg/dl	189 ± 5.9	192 ± 5.8	195 ± 8.7	NS
HTN	60	56	15	NS
Family history of CAD	45	83	8	0.004
Diabetics	30	16	5	NS

Data is presented as mean± SD, descriptive variables are presented as number of subjects NS= non-significant, p values for chi-square analysis less than 0.05 is significant

**Table-IV:** Multivariable linear regression analysis between Val109Asp SNP and CAD.

*Model*	*Genotype*	*Cases (n = 250)*		*Controls (n = 100)*		*OR (95% CI)^a^*	*p*	*Adjusted OR(95% CI)^b^*

		*n*	*%*	*n*	*%*			
Dominant	AT	147	58%	30	30%	1.000 (Reference)		1.000 (Reference)
	TT + AA	103	42%	70	70%	2.782(1.861-4.159)	0.02	2.988(1.717-5.202)
Recessive	TT + AT	169	68%	37	37%	1.000 (Reference)		1.000 (Reference)
	AA	81	32%	63	63%	7.473(2.430-22.983)	0.04	9.205 (2.336-36.280)

Using multivariable regression analysis; adjusted for age, gender, BMI, waist circumference smoking, hypertension, diabetes mellitus and lipid abnormalities. Odds ratio (95% confidence interval) was expressed for the risk of the other genotype when AT or TT+AA genotype was referenced.

## DISCUSSION

The present study has established that omentin-1 gene Val109Asp (326 +A/T) missense polymorphism is frequently present in both groups of current population, However, Homozygous genotype Asp/Asp was more often in control subjects (OR= 0.335; 95% CI 0.2241-0.5635), whereas CAD patients were detected with increased heterozygous mutant genotype Val/Asp (OR=1.921; CI 1.173-3.1469). The current study had indicated that the ‘T’ allele at position 326 in exon 4 of omentin-1 gene acts as a dominant allele in relation to increased risk of CAD while, homozygous genotype Asp/Asp reduces the risk of developing CAD (Table-II). To the best of our knowledge there is a single study in literature by Yörük et al. who investigated the relatioship of omentin-1 gene Val109Asp polymorphism and CAD in Turkish population but found no significant association reason might be recruitment of small number of CAD patients, however, it was observed that Val/Val heterozygous genotype was more frequent in CAD patients.^12^ We analyzed omentin-1 gene Val109Asp polymorphism in CAD patients in Pakistani population and found the significant difference between cases and controls regarding this SNP in terms of genotype distribution, although we could not find any significant association between Val109Asp polymorphism and standard clinical, and metabolic factors. Subjects reported with strong family history of CAD had been identified with more heterozygous genotype Val/Asp. Male predominance with this SNP had also been observed in current study (Table-III). By multivariable regression analysis, our data showed that AT genotype still remained the strong association with HU after adjustment for some well-known CAD risk factors included age, gender, BMI, waist circumference, smoking, blood pressure, blood glucose, cholesterol and diet, suggesting Val109Asp might be an independent risk factor for CAD susceptibility (Table-IV).

It has been documented by various resent studies that the adipose tissue is not just a fat deposit but it also acts as an endocrine tissue and produces numerous hormone like proteins called adipokines including: leptin, adiponectin, vaspin, TNFa, IL 6, IL 10, resistin, visfatin and omentin.^13^ Visceral adipose tissue is dynamically involve in inflammation and its hypertrophy is associated with pathogenesis of various disorders like diabetes, metabolic syndrome, arthritis and CAD.^14^ Omentin-1 is newly identified protein secreted by omental visceral fat. It has metabolic effects on myocytes, hepatocytes and adipocytes. It acts as an anti-inflammatory protein via nuclear factor Kappa b(NFkb) pathway.^15^ The location of omentin-1 gene is q22-q23 of chromosome 1, this location has found to be linked with Type-2 diabetes mellitus in some populations. In-vitro experiments have been shown that omentin-1 recombinant therapy enhanced glucose uptake by cells via Akt signaling pathway^16^,^17^, moreover, plasma concentration of omentin-1 and its mRNA expression in VAT is found to be reduced in obesity and diabetes.^18^ Some studies have made known the correlation between obesity, insulin resistance adipokines and pathogenesis of CAD.^19^,^20^ Yamawaki et al. had demonstrated that omentin-1 may directly induced vascular relaxation via inhibition of endothelial nitric oxide synthase (eNOS).^21^ As omentin-1is highly expressed by epicardial fats, so it might has protective role in coronary artery atherosclerosis and obesity related cardiac events.^22^ After considering these outcomes that serum omentin-1 circulating levels are significantly associated with coronary artery atherosclerosis, it seems reasonable to investigate omentin-1 gene polymorphism and its correlation with of CAD in pakistani population. Although, several disease like psoriasis^23^, diabetes^14^, rheumatoid arthritis^24^ had been investigatd in association with Val109Asp SNP but found no significant correlation.

In current study CAD patients were recruited from three different centers of Karachi, belong to different ethinic groups. The common factor that observed was central obesity even with normal BMI. As omentin-1 expressed from visceral fats so it seem reasonable to investigate the omentin-1 polymorphism and coronary artery disease. So the data from this study will help the researchers to investigate the pathophysiology behind the association between polymorphism and CAD, which is still unknown.

Current study made evident that there is a significant association between omentin-1 polymorphism and risk of developing coronary artery disease.

## CONCLUSION

This is perhaps the first study in Pakistan that has established a significant association between omentin-1 Val109Asp polymorphism and coronary artery disease. It also revealed that heterozygous Val/Asp genotype of omentin-1 gene is more associated with risk of developing CAD in Pakistani population while healthy subjects were found with more Asp/Asp genotype, however studies with different ethnicities are required to confirm our findings.

### Rationale of study

Our study, to best of our knowledge, is the first study in Pakistan that established the association of omentin-1 gene with coronary artery disease. This study not only revealed the genetic aspect of coronary artery disease but also provide a local data which help in future studies.
